# Perceived school service quality and vocational students’ learning satisfaction: Mediating role of conceptions of vocational education

**DOI:** 10.1371/journal.pone.0307392

**Published:** 2024-08-21

**Authors:** Tao Guo, Tianxin Li, Zhanyong Qi

**Affiliations:** Faculty of Education, Shaanxi Normal University, Xi’an, China; Beijing University of Technology, CHINA

## Abstract

This study examined the relationships among vocational students’ perceptions of school service quality, their learning satisfaction, and their conceptions of vocational education in Chinese secondary vocational schools. Using a quantitative approach, data were collected from 10,721 students through multistage sampling. Perceived school service quality was assessed using the five-factor SERVPERF instrument, learning satisfaction was measured with the one-factor SSwLA scale, and conceptions of vocational education were evaluated using the one-factor SCoVE scale. These instruments were subjected to internal, convergent, discriminant, and construct validity tests, including exploratory and confirmatory factor analyses. Structural equation modeling (SEM) analyzed the relationships among the constructs. Additionally, mediation analysis was employed to explore the mediating role of students’ conceptions of vocational education in the relationship between perceived school service quality and learning satisfaction. Results indicated that learning satisfaction was positively influenced by students’ perceptions of school service quality, particularly responsiveness, assurance, reliability, and empathy, but negatively by tangibles. Furthermore, the association between students’ perceived school service quality and learning satisfaction was mediated by their conceptions of vocational education, highlighting the complex interaction between perceived service quality and students’ learning satisfaction. These findings provide critical insights for policymakers and educators seeking to enhance effectiveness and satisfaction within vocational education settings.

## Introduction

Understanding the quality of educational services within vocational sectors is pivotal for enhancing educational outcomes and aligning with government priorities [1, 2]. The Chinese government’s emphasis on elevating the professional caliber and skill set of its workforce, as highlighted in the report to the 18th National Congress of the Communist Party of China [3] and reinforced by the newly revised Vocational Education Law of 2022 in China [4], underscores China’s commitment to modernizing vocational education [5–7]. This legislative and policy framework aims to not only bolster the nation’s technical proficiency but also to elevate vocational education to a status commensurate with general education, thereby addressing the demands of new industrialization and societal advancement.

However, societal perceptions have historically undervalued vocational education in China, regarding it as inferior to general education [8, 9]. Poor quality of service offered by vocational schools is a contributing factor to the lack of trust and dissatisfaction with vocational education in China among the public [10, 11]. Vocational education in China has been long criticized for its weak teaching quality [12], low wage income and social status after graduation [9]. Challenges within vocational education are not confined to China, as the relatively low status of vocational education and training pervades both developing and advanced industrial nations [13]. International studies highlight similar challenges: vocational students in Indonesia report online learning as insufficient for skill development [14], while research in Malaysia points to obstacles like student motivation, societal biases, and resource limitations [15]. Additionally, studies from Germany and Australia indicate a broader need to reconsider vocational education’s positioning and the influence of familial and educational guidance on career choices [16]. These insights collectively suggest an urgent need to refine vocational education strategies by addressing the diverse array of attitudes and experiences that hinder its perception and effectiveness.

In response to these issues, this study aims to address the following research questions:

1. How do Chinese secondary vocational students perceive school service quality and vocational education within China?
2. How do students’ perceptions of school service quality and their conceptions of vocational education influence their satisfaction with learning achievements?

Through an in-depth examination of student perceptions and their correlation with learning satisfaction, this research endeavors to illuminate the factors that contribute to learning satisfaction within the context of vocational education. The findings from this analysis are designed to provide valuable insights that can guide policy-making and educational practices, thereby improving the quality of vocational education and elevating its status both nationally and globally.

## Theoretical background and hypotheses’ development

### Service quality theory and models

The exploration of service quality in the educational sector, initially rooted in business and marketing research, underscores the vital role of meeting customer needs and expectations [17]. Within this domain, the SERVQUAL and SERVPERF models have emerged as prominent tools for evaluating service quality [18]. SERVQUAL identifies five core dimensions of service quality—tangibles, reliability, responsiveness, assurance, and empathy—offering a comprehensive framework for assessment [19]. In contrast, SERVPERF adapts SERVQUAL by focusing solely on service perceptions to mitigate the model’s challenges with expectations, which has been a subject of debate regarding its interpretability and operationalizability.

The application of these models across educational settings reveals their versatility and adaptability, as noted by Parasuraman et al. [20]. However, the models’ application has not been without criticism. For instance, the expectation component in SERVQUAL has been questioned for its variability among individuals, potentially complicating the assessment of service quality. SERVPERF’s elimination of the expectation measure simplifies the model but at the risk of overlooking an essential aspect of service evaluation.

Empirical adaptations and applications of these models in various educational contexts have demonstrated both their strengths and limitations. Yildiz and Kara [21] introduced the PESPERF scale, a variant of SERVQUAL, which pinpointed academic aspects, empathy, and access as critical to service quality in educational settings in Turkey, suggesting the need for model customization to specific contexts. This contrasts with findings from Hussain and Birol [22] and others who reported a uni-dimensional structure of service quality in North Cyprus, indicating a variance in the significance of SERVQUAL’s dimensions across contexts. Further, the utilization of SERVQUAL by Oliver and Koeberg [23] in the Western Cape and the development of the EDUSERV model by Ramseook-Munhurrun and Nundlall [24] in Mauritius underline the model’s flexibility but also its variable applicability depending on contextual factors such as cultural and institutional settings. These adaptations highlight the ongoing debates around the SERVQUAL model’s dimensionality and the necessity for its modification to align with specific educational needs and goals.

Additionally, the SERVQUAL model’s application across Asian educational contexts reveals its adaptability yet underscores its limitations. Studies like El-Hilali et al. [25] in Kuwait and Uppal et al. [26] in Pakistan affirm the model’s utility, identifying key dimensions such as tangibility and responsiveness as significant to student satisfaction. However, variations in the model’s effectiveness are noted, with Alharbi et al. [27] observing discrepancies in service quality perceptions at Saudi conferences, and He [28] adapting the model for Chinese universities to highlight the importance of four specific dimensions. Additionally, Yang and Mao [29]’s development of an internship quality index in China further validates SERVQUAL’s applicability, while Li [30]’s study on online learning dissatisfaction points to the model’s challenges in addressing all facets of educational quality. These insights collectively highlight SERVQUAL’s broad relevance in assessing service quality within education, yet call for contextual adjustments to fully capture the diverse aspects of student satisfaction.

Summarizing the application of SERVQUAL and SERVPERF in educational service quality assessments reveals a consistent framework across various contexts: tangibles, reliability, responsiveness, assurance, and empathy [31, 32]. Despite adaptations to meet specific educational environments, these five dimensions fundamentally underpin service quality evaluation, highlighting physical infrastructure, instructor competency, administrative support, system trustworthiness, and empathetic teacher-student interactions. The present study aims to explore these dimensions within Chinese secondary vocational education, assessing their relevance and effectiveness in this unique setting. Hence, we hypothesize the following:

**H1**: **Chinese students in secondary vocational education assessed the service quality of their school using five criteria: tangibles, reliability, responsiveness, assurance, and empathy**.

This hypothesis is grounded in a critical analysis of the theoretical frameworks discussed, reflecting a thorough understanding of their strengths and limitations, and the importance of contextualizing these models to enhance their utility and relevance in assessing educational service quality.

### Students’ satisfaction with learning achievements

Satisfaction is the sensation a person has when his or her needs are met or when one’s expectations are met [33]. At present, higher education institutions are placing a larger emphasis on student satisfaction because it is an important indicator of school effectiveness in regard to a set of student expectations [34]. Scholars have conducted extensive and in-depth research on student satisfaction, encompassing a variety of academic stages (junior/senior middle school, vocational training, and adult education) and topics, such as teaching quality [35], training service [36], campus life [37], housing facilities [38], technology in educational settings [39] and virtual learning environments [40].

Students’ satisfaction with their learning achievement (SSwLA) is the outcomes of their academic experiences. It has been found that the positive student-teacher relationship contributed to students’ achievements [41], and these attainments include both students’ skills and knowledge [42] and career advancement [43]. It has also been proven that faculty performance and classes are positively related to the outcome of college experience and achievements. In addition to faculty performance, advisory staff play critical roles in student achievements because students will be dissatisfied if these roles are not fulfilled. According to Letcher and Neves [44], various service quality characteristics had an impact on students’ intellectual performance. Rapert et al. [42] came up with ideas about quality like process characteristics and outcome attributes to explain how teaching, advising, friendly interactions between staff and students, and programs positively influenced students’ achievements.

In addition to these aspects, self-determination theory (SDT) provides an extensive explanation of how motivation impacts students’ learning satisfaction. SDT posits that satisfaction in educational environments can be greatly enhanced by fulfilling students’ needs for autonomy, competence, and relatedness [45]. Autonomy refers to the student’s ability to have control over their learning processes; competence involves feeling proficient in their academic pursuits; and relatedness denotes a sense of connection with teachers and peers [45]. These factors inherently motivate students, leading to higher intrinsic motivation which is closely linked to deeper engagement and satisfaction with learning outcomes [46]. Moreover, empirical studies have shown that intrinsic motivation, fostered through meeting these three basic needs, leads to higher educational satisfaction compared to extrinsically motivated behaviours which are driven by external rewards or pressures [47]. For instance, when students perceive their learning environment as supportive of their autonomy, they are more likely to engage deeply in their studies, thus experiencing higher satisfaction from their achievements [48, 49].

This literature suggests that various aspects of educational service—ranging from teaching quality to the efficacy of advisory staff—play a pivotal role in shaping student satisfaction. These aspects, which are integral to the broader construct of service quality, have been shown to influence students’ academic experiences, skills, knowledge acquisition, and career progression positively. Such evidence bolsters the hypothesis by demonstrating a direct link between superior educational service quality and enhanced student learning satisfaction.

The importance of positive student-teacher interactions in fostering student achievements suggests that relational dimensions of service quality—captured by responsiveness and empathy in the SERVQUAL model—crucially meet students’ expectations and boost their satisfaction [42]. The initiation of the China Customer Satisfaction Index by Tsinghua University [50], coupled with government initiatives emphasizing quality and satisfaction in services, including education, highlight the national significance of these concepts [51]. However, the scarcity of focused research on satisfaction within secondary vocational and technical education signals a gap this study intends to fill. Hence, based on the theoretical and empirical evidence between the variables, we hypothesize the following:

**H2**: **SPoSSQ has a positive relationship with students’ satisfaction with learning achievements (SSwLA)**.

### Students’ conceptions of vocational education

Students’ conceptions of vocational education (SCoVE) have a significant impact on their emotions and behavior [52]. Terms such as beliefs, conceptions, perceptions, and attitudes are used to describe what students think, believe, and feel [53, 54] and are associated with students’ cognitive understanding and evaluations, as well as their emotional responses to their learning tasks [55]. Consequently, students’ satisfaction with their learning outcomes in vocational education and training is heavily influenced by their attitude toward vocational education and training. Moreover, students’ attitudes can significantly impact their perceptions of the value of vocational education and training and their eventual participation in such programs [56]. Hence, students’ conceptions of vocational education are considered a dependent variable that is impacted by their perceptions of the service quality in vocational education and training.

Attitudinal theories posit that attitudes are formed through experiences and can be reshaped by processes of perception and reflection [57]. This notion suggests that improvements in the quality of service within vocational education have the potential to positively influence students’ attitudes toward their schooling, which in turn could elevate their levels of satisfaction. This relationship is underpinned by evidence demonstrating that comprehensive quality of service in education—including factors like instructional quality and support services—plays a critical role in heightening students’ overall satisfaction with their educational experience [58]. Furthermore, it is imperative to acknowledge the influence of external societal and economic factors on student satisfaction. These factors indicate that the relationship between service quality and satisfaction is not straightforward but is instead likely to be mediated by external variables. This complexity highlights the multifaceted nature of educational satisfaction. Therefore, it is hypothesized that:

**H3**: **Students’ conceptions of vocational education (SCoVE) mediate the effects of SPoSSQ on students’ satisfaction with their learning achievement (SSwLA)**.

The literature analysis yields three primary hypotheses that relate school service quality in vocational education (SPoSSQ) to students’ satisfaction with learning outcomes (SSwLA), with a focus on the mediating function of students’ conceptions of vocational education (SCoVE). This study of the direct and indirect effects of perceived service quality on student learning satisfaction shows an underexplored topic, particularly in the context of Chinese vocational education. It emphasizes the importance of students’ perspectives of their educational experiences in developing these connections. This study intends to broaden the discussion on education quality by investigating these assumptions and providing practical insights for improving service quality and students’ educational experiences and accomplishments in vocational contexts.

## Materials and methods

### Research design

**Fig 1:**
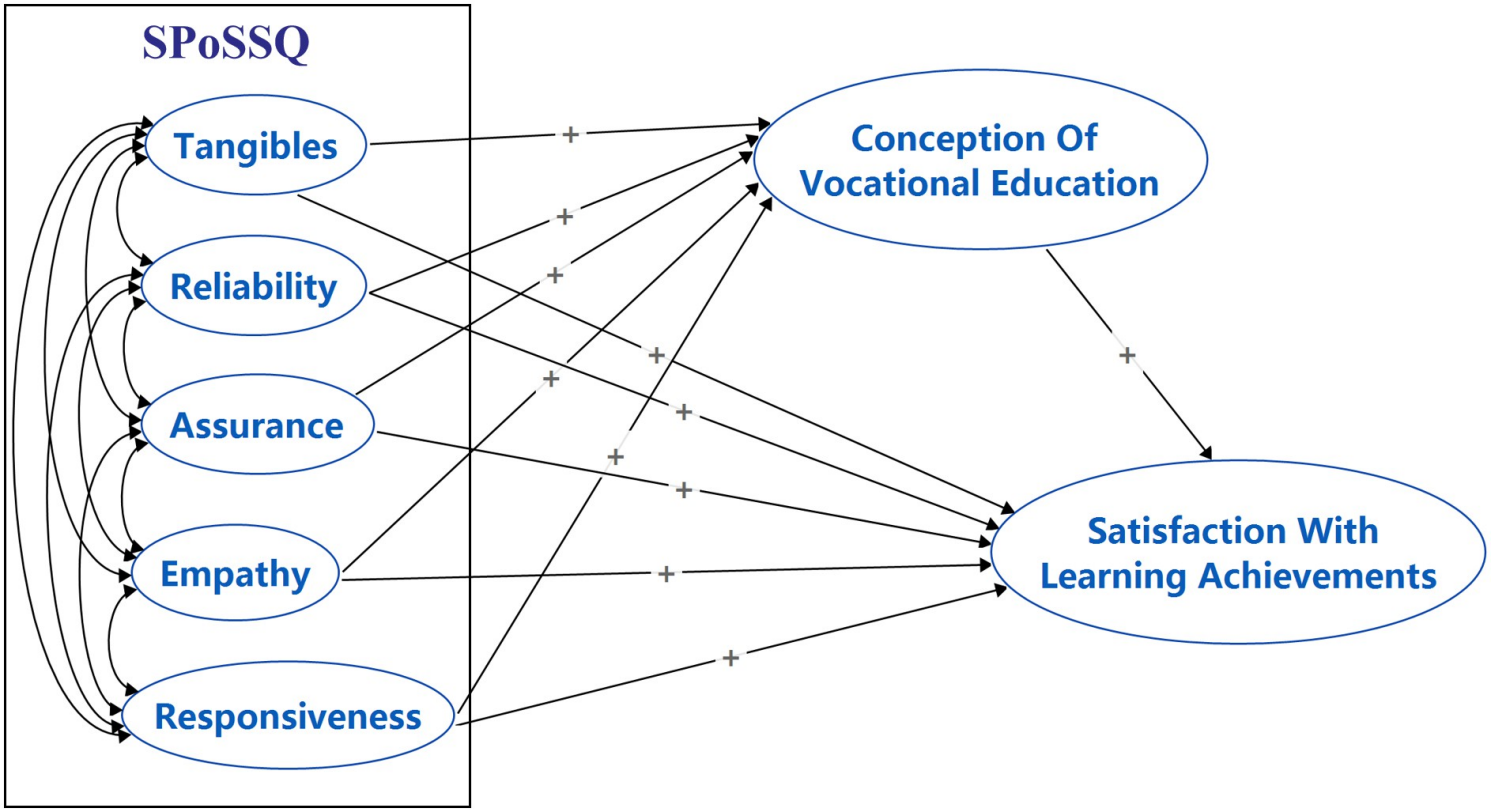
Conceptual framework of the current study. The "+" symbol indicates a positive influence or association between the connected variables.

Based on the literature and the three mentioned hypotheses, the conceptual framework (Fig 1) for the study is established. Fig 1 shows that five factors in SPoSSQ, namely: tangibles, reliability, assurance, empathy, and responsiveness, will have a direct positive relationship with SSwLA. Besides, five factors in SPoSSQ will positively predict SSwLA through SCoVE. The current study employed a quantitative research approach. This research was aimed at studying the structure of SPoSSQ in Chinese secondary vocational schools and the relationship between SPoSSQ, SSwLA, and their conceptions of vocational education (SCoVE).

#### Participants

**Fig 2:**
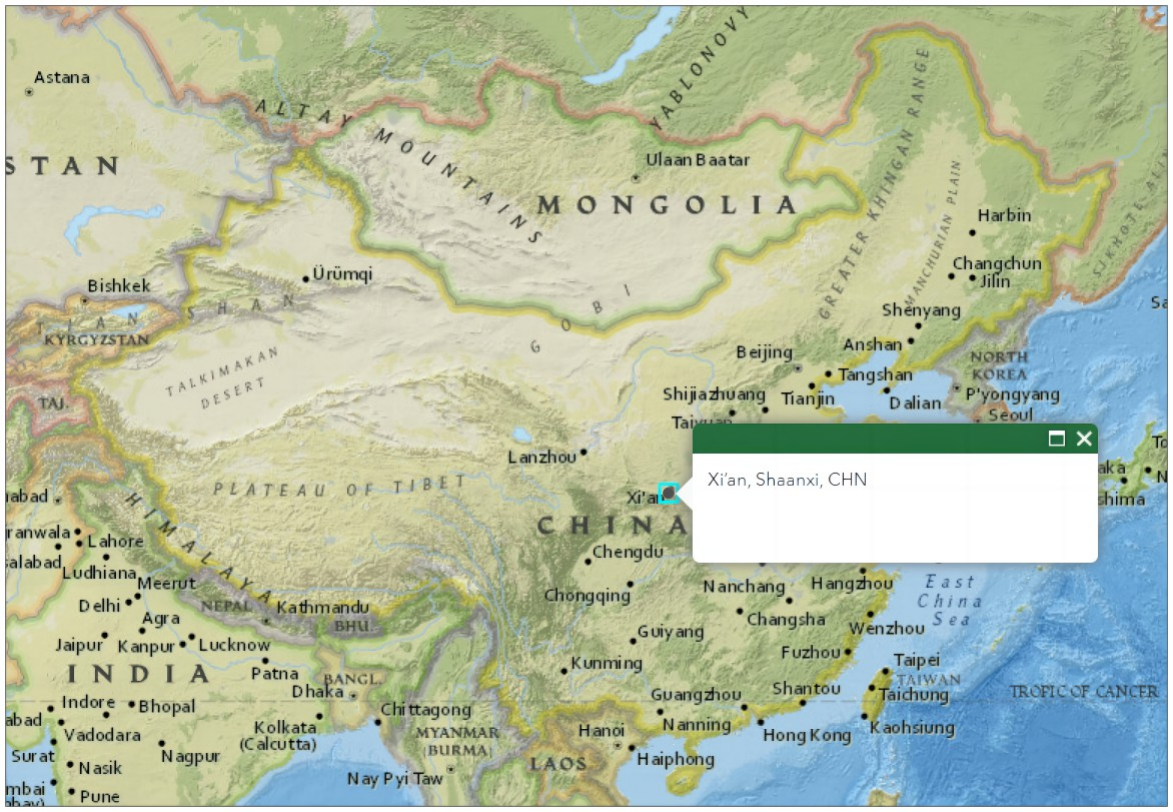
Study site in Xi’an, Shaanxi, China. The map is sourced from the USGS National Map Viewer.

The participants in this study were a subgroup of Chinese secondary vocational students in Xi’an City (n = 10,721) who were part of the Basic Education Enhancement Three-Year Action Plan Program in Xi’an. This program, initiated by the local educational authorities in Xi’an (as shown in Fig 2), aims to elevate the standard of basic education by integrating advanced pedagogical methods, improving infrastructure, and enhancing teacher training within secondary vocational schools. By focusing on students enrolled in this program, the study taps into a segment of the vocational student population that is directly experiencing these educational enhancements.

This figure illustrates the geographic area of Xi’an, Shaanxi, China, indicating the study sites where data were collected for the research. The figure was sourced from the USGS National Map Viewer, a public domain resource that offers comprehensive mapping data and visuals (https://apps.nationalmap.gov/viewer/). To ensure a comprehensive representation of the student body, recruitment was strategically executed through a purposeful stratified random sampling technique. This approach was particularly vital given Xi’an City’s diverse landscape of vocational education, characterized by schools varying in size, specialization, and location across urban and rural areas. Initially, the population was divided into distinct subgroups or strata based on key characteristics such as school size, specialization, and geographic location. Within each stratum, participants were randomly selected to ensure adequate representation of each subgroup in the sample. To further enhance the relevance of the sample, purposive sampling measures were employed to include students who were actively engaged in the educational enhancements provided by the Basic Education Enhancement Three-Year Action Plan Program. This dual approach allowed the study to focus on students most likely to provide valuable insights into the impact of these enhancements on their educational experiences, while also ensuring that the diverse landscape of vocational education in Xi’an was adequately represented. During the research design process, an A-priori Sample Size Calculator for Structural Equation Models [59] recommended a minimum sample size of 1,808 participants when the numbers of latent and observed variables are 7 and 40, respectively, and the power level = 0.8. Hence, the sample size was sufficient for the present study.

Inclusion criteria were precisely defined to include students actively participating in secondary vocational programs associated with the Basic Education Enhancement Three-Year Action Plan Program. Conversely, exclusion criteria were applied to exclude students who either did not provide informed consent or were outside the scope of the target educational initiatives. This careful delineation of participant criteria, combined with the methodical sampling approach, ensures that the study’s outcomes can be considered representative of the diverse vocational student population in Xi’an.

Students’ perceptions of school service quality, their conceptions of vocational education, and their satisfaction with learning achievements were assigned to a subset of the program questionnaire, which was randomly assigned to secondary vocational students participating in the program’s online questionnaire. Several measures were implemented to ensure the quality of data collection. The online questionnaire was designed to prevent multiple submissions from the same participant and included validation checks to ensure completeness and accuracy of responses. Participants were given clear instructions and encouraged to answer all questions honestly. Regular monitoring of response rates and patterns was conducted to identify and address any anomalies promptly. The effectiveness rate of the questionnaire was high, with a completion rate of 93%, indicating strong engagement and reliability of the data collected. This high response rate, coupled with rigorous data quality measures, ensured that the findings of the study are robust and representative of the diverse vocational student population in Xi’an. Extensive measures were implemented to safeguard the privacy and integrity of the data provided by the participants. Informed consent was electronically obtained from participants and their guardians after providing comprehensive study information, emphasizing anonymity and data security. The study, which received ethical approval from the Research Ethics Committee of the School of Education at Shaanxi Normal University, ensured no identifiable information was collected, maintaining strict confidentiality and data protection.

The commencement of data acquisition for the current study was marked on the 15th of March, 2022 and reached its conclusion on the 1st of July, 2022. The demographic constitution of the sample population was such that it predominantly consisted of female students, accounting for 57.2% of the total, whilst their male counterparts represented the remaining 42.8%. The sample size included 5,431 Year 1 students, 4,277 Year 2 students, and 1,013 Year 3 students. Additionally, more than half of the participants were students from state-funded schools (n = 8,068), while the remaining proportion consisted of students enrolled in private schools (n = 2,653). Comprehensive participant demographics are provided in Table 1.

**Table 1:** Demographic characteristics of questionnaire participants.

Characteristics		N	Valid %
Gender	Male	4,588	42.8
	Female	6,133	57.2
School enrolled	State-funded school	8,068	75.3
	Private school	2,653	24.7
Year Level	1st year	5,431	50.7
	2nd year	4,277	39.9
	3rd year	1,013	9.4
Household registration location	City	1,773	16.5
	County	706	6.5
	Rural area	8,047	76.9
Family monthly income	Very wealthy	909	8.5
	Wealthy	3,379	31.5
	Ordinary	6,275	58.5
	impoverished	141	1.3
	Very impoverished	17	.2

#### Measures

This study employed a two-part anonymous questionnaire, with Part 1 consisting of demographic inquiries that encompassed individual, household, gender, and geographical factors. Part 2 of the study incorporated validated measurement items, including the SPoSSQ scale, the SSwLA scale, and the SCoVE scale, all tailored for the Chinese secondary vocational education context. The details of constructs and their corresponding measurement items are presented in S1 Appendix. Content validity was thoroughly verified by soliciting reviews from two university educators with expertise in the Chinese educational system. Their assessments focused on the relevance, breadth, and accuracy of the constructs measured, leading to refinements that enhanced construct representation. Additionally, face validity and reliability were confirmed through a pilot test involving 80 university students, resulting in minor modifications to the questionnaire to improve its clarity and relevance.

*Students’ Perceptions Of School Service Quality Scale (27 items*, *Cronbach’s α* = .*98)*. The Students’ Perceptions of School Service Quality Scale (SPoSSQ), adapted from Nadiri et al. [60], was utilized to gauge students’ perceptions of service quality in Chinese vocational schools. Rated on a five-point Likert scale from 1 (strongly disagree) to 5 (strongly agree), the SPoSSQ allows for a detailed exploration of student perceptions across different dimensions of service quality, namely tangibles, reliability, responsiveness, assurance, and empathy. The scale’s total score, ranging from 27 to 135, serves as a comprehensive indicator of perceived service quality, with higher scores denoting increased satisfaction.

Modifications to the original scale were validated for reliability and validity through extensive analysis, including Cronbach’s alpha to ensure internal consistency, with the pilot study revealing alpha values of 0.98 overall and high reliability across dimensions (0.96 for tangibles, 0.95 for reliability, 0.89 for responsiveness, 0.93 for assurance, and 0.91 for empathy). Additionally, in the analysis process, convergent and discriminant validity were confirmed via factor loading, composite reliability (CR), and average variance extracted (AVE), alongside exploratory and confirmatory factor analyses to establish construct validity. These measures affirm the modified scale’s effectiveness in providing reliable and valid assessments, capturing the nuanced perceptions of vocational school students with high fidelity.

*Students’ satisfaction with learning achievements scale (8 items*, *Cronbach’s α = 0*.*97)*. The student satisfaction with learning achievements scale, drawing on Topala and Tomozii [61], is a concise 8-item tool designed to evaluate students’ contentment with their academic and non-academic achievements during their schooling. It boasts a Cronbach’s alpha of 0.97, indicating high reliability. Responses are gathered using a five-point Likert scale (1 = strongly disagree, 5 = strongly agree), enabling a nuanced understanding of student satisfaction. The total possible scores range from 8 to 40, with higher scores reflecting greater satisfaction levels. Convergent and discriminant validity of the modified scale were established through factor loading, composite reliability (CR), and average variance extracted (AVE), complemented by exploratory and confirmatory factor analyses to ensure construct validity, collectively affirming the scale’s capacity for reliable and valid evaluations of vocational school students’ perceptions.

*Students’ Conception of Vocational Education Scale (7 items*, *Cronbach’s α = 0*.*97)*. The Students’ Conception of Vocational Education Scale, refined from Kilasa et al., [62], is precisely adapted to the nuances of Chinese secondary vocational education. It includes 7 items and boasts a high Cronbach’s alpha of 0.97, indicating excellent reliability in measuring students’ perceptions and attitudes towards vocational education policy and their confidence in vocational pathways. This scale is designed to elucidate students’ beliefs about the role and effectiveness of vocational education in advancing their career prospects. Utilizing a five-point Likert scale for responses (ranging from 1 = strongly disagree to 5 = strongly agree), the scale offers a detailed view of students’ levels of agreement or satisfaction. Scores can vary between 7 to 35, where higher scores signify increased satisfaction and positive perceptions of vocational education.

#### Statistical analysis

To ensure the quality of the survey instrument, this study rigorously validated the scales used to assess students’ perceptions of school service quality, satisfaction with learning achievements, and conceptions of vocational education. Validation was meticulously conducted for SPoSSQ, SSwLA and SCoVE using the SPSS V.27 and AMOS V.26 software to ensure the reliability and validity of the measurements. Internal consistency for each scale was evaluated using Cronbach’s alpha, providing a measure of the cohesiveness of the items within each scale. Convergent and discriminant validity of SPoSSQ, SSwLA and SCoVE were assessed using factor loading, composite reliability (CR), and average extracted variance (AVE). Additionally, exploratory and confirmatory factor analysis were conducted to evaluate the construct validity of SPoSSQ, SSwLA and SCoVE, which assessed how effectively a set of indicators represented a concept that may not be immediately quantifiable.

A combination of exploratory factor analysis and confirmatory factor analysis was used in two stages to analyze the data [63]. The first stage of the analysis involved determining the measurement models of SPoSSQ, SSwLA and SCoVE through exploratory factor analysis, which employed maximum likelihood extraction and oblique rotation, as recommended by Costello and Osborne [64]. Following confirmation of the measurement model, the second stage involved modelling the hypothesized theoretical causal relationships between the latent factors in SPoSSQ, SSwLA and SCoVE using structural equation modelling (SEM) [65].

In accordance with literature [66, 67], the fitness of the model of SPoSSQ, SSwLA, SCoVE and the hypothesized theoretical model in the current study was determined by two indices: a goodness-of-fit measure and a badness-of-fit measure, which suggested that models should be accepted if 1) the comparative fit index (CFI) and gamma hat >.90; and if 2) the root mean square error of approximation (RMSEA) and the standardized root mean residual (SRMR) < .08. In relation to stage one, if the theoretical models fit the data poorly or not at all, modifications to the model were needed to resolve the issues. In the current study, such modifications included merging highly correlated factors, using modification indices (MI) to identify items that were highly attracted to multiple factors or to other items, and fixing negative error variances to a small positive value (i.e.,.005) if twice the standard error was bigger than the estimated variance [68]. Subsequently, an array of trimming principles was applied to deal with recursive and admissible models with poor fit. To improve the model fit in the present study, the statistically non-significant paths and items, and items with weak loading (i.e.,.30) on their respective factors, were removed [69]. Other strategies included deleting items that had a high attraction to logically inappropriate factors according to their MI and collapsing factors with fewer than three items.

## Results

### Measurement models

#### Students’ perceptions of school service quality (27 items)

The initial exploratory factor analysis revealed five factors that explained 85.70% of the total variance. The Bartlett test of sphericity was significant (p<0.05), indicating that the variables were intercorrelated. The Kaiser-Meyer-Olkin (KMO) measure of sampling adequacy was computed to assess the degree of intercorrelations among the variables, yielding an index of 0.987. This value indicates that the use of factor analysis was appropriate, as the KMO measure of sampling adequacy exceeded the recommended threshold of 0.6. Convergent validity was established, as all factor loadings exceeded 0.5. Furthermore, Cronbach’s alpha values ranged from 0.97 (maximum) to 0.94 (minimum), indicating high internal consistency reliability.

To identify the best fit factor structure for Chinese secondary vocational students, confirmatory factor analysis was conducted on three models: one inter-correlated model, one hierarchical model, and one bi-factor model. These models were evaluated to determine the optimal factor structure, and included (1) the theoretically hypothesized five-factor model (Model 1); (2) a hierarchical model with five sub-factors (Model 2); and (3) a five-factor bi-factor model (Model 3), which incorporated a general factor into the five-factor model. Of the three models evaluated, only Model 3 failed to demonstrate an acceptable fit to the data. Table 2 presented the fit indices for the two remaining competing models used to determine the factor structure of the SPoSSQ for Chinese students. Based on the results, Model 1 was selected as the best fitting model, as indicated by its smaller AIC value (Δ = 5232.33) compared to that of Model 2.

**Table 2:** SPoSSQ model fit indices.

Model	Fit indices									
	k	*X* ^2^	*df*	*X*^2^/*df*	*p* value	CFI	gamma hat	RMSEA (90% CI)	SRMR	AIC
Model 1	27	13496.37	314	42.98	< .000	.97	.92	.063 (.062-.063)	.021	13678.37
Model 2	27	18738.70	319	58.74	< .000	.96	.89	.073 (.072-.074)	.037	18910.70

*Note*. K = Number of items; CFI = Comparative fit index; RMSEA = Root mean square error of approximation; CI = Confidence interval; SRMR = Standardized root mean residual; AIC = Akaike information criterion; * = preferred model.

In Model 1, all variables exhibited normal distribution characteristics, as evidenced by skew and kurtosis values falling within acceptable ranges (skew = 3.00, kurtosis = 7.00). The SERVPERF model, consisting of five factors as theorized, was successfully replicated in this model. To evaluate the model’s fitness and test hypotheses, as well as establish construct reliability and validity of the measurement model, covariance-based structural equation modeling (CB-SEM) was performed using SPSS V.27 and AMOS V.26. The validity and reliability of the constructs were assessed using confirmatory factor analysis (CFA) through the maximum likelihood method.

The assessment of convergent and discriminant validity was conducted using factor loading, composite reliability (CR), and average extracted variance (AVE). In general, a factor loading exceeding 0.70 is considered substantial, and the removal of items with factor loading between 0.40 and 0.70 is only recommended if doing so would improve the CR or AVE score [70]. As demonstrated in Table 3, all estimates of CR and AVE surpassed the predefined cutoff criteria, confirming the validity and convergent nature of the measurement model. These results indicate that the measurement model was both reliable and valid.

**Table 3:** SPoSSQ factor means, SDs, Cronbach’s alpha, CR, AVE, and effect size.

Factors	#Items	M	SD	Alpha	CR	AVE	Cohen’s *d*				
							1	2	3	4	5
1. Tangibles	8	3.50	.849	.958	.959	.744	-				
2.Reliability	4	3.72	.806	.941	.961	.859	.11	-			
3.Responsiveness	5	3.59	.843	.960	.942	.764	**.27**	.16	-		
4.Assurance	5	3.63	.824	.974	.974	.883	.16	.05	-.11	-	
5.Empathy	5	3.77	.809	.971	.971	.871	**.33**	**.22**	.06	.17	-

*Note*. M = Mean; SD = Standard deviation; Values of Cohen’s effect sizes shown in italics below diagonal.

The mean scores (Table 3) of factors in this correlated model demonstrated that Chinese secondary students in vocational education agreed with the assumption that school service quality was concerned with tangibles, reliability, responsiveness, assurance, and empathy, which supported the H1. The mean scores for all five factors were over 3.50, indicating that students were usually impressed with all five areas of service quality in their school. The highest score resided in empathy (M = 3.77) while the lowest mean score was 3.50 in tangibles. After examining the effect size for the difference between all criteria, it was possible to conclude that participants had more positive attitudes about empathy than tangibles or reliability, and that they were more satisfied with responsiveness in their school than the tangibles variable.

#### Students’ satisfaction with learning achievements scale (6 items)

One factor referred to students’ achievement satisfaction (*a* = 0.97) and was extracted and accounted for 90.66% of the total variance. The confirmatory factorial analysis was not applicable because only one factor was extracted.

#### Students’ conceptions of vocational education (7 items)

One factor referred to students’ conceptions of vocational education (*a* = 0.98) and was extracted and accounted for 82.02% of the total variance. The confirmatory factorial analysis was not applicable because only one factor was extracted.

### Structural equation modelling

To test the H2 and H3, implying the relationships among the three constructs (SPoSSQ, SSwLA, and SCoVE), a systematic testing of possible pathways was carried out using Amos V.26. Model 1 was the baseline model, where all constructs were independent of each other. Model 2 was developed with reference to the theory of service quality, where the SPoSSQ predicted the SSwLA through the SCoVE and the SCoVE acted as a mediator. Table 4 provided the model fit index for these two models. As only Model 2 achieved acceptable model fit indices and the AIC value of Model 2 was the lowest, Model 2 was chosen as the best fit to the data.

**Table 4:** Model fit indices of two competing models.

Model		Fit Indices								
	k	χ^2^	*df*	χ^2^/*df*	*p* value	CFI	gamma hat	RMSEA (90% CI)	SRMR	AIC
Model 1	40	61960.46	730	84.87	< .001	.91	.78	.088 (.088–.089)	.506	62220.43
Model 2	40	23129.52	719	32.17	< .001	.97	.90	.054 (.053–.054)	.026	23411.52

*Note*. K = Number of items; CFI = Comparative fit index; RMSEA = Root mean square error of approximation; CI = Confidence interval; SRMR = Standardized root mean residual; AIC = Akaike information criterion; * = preferred model.

**Fig 3:**
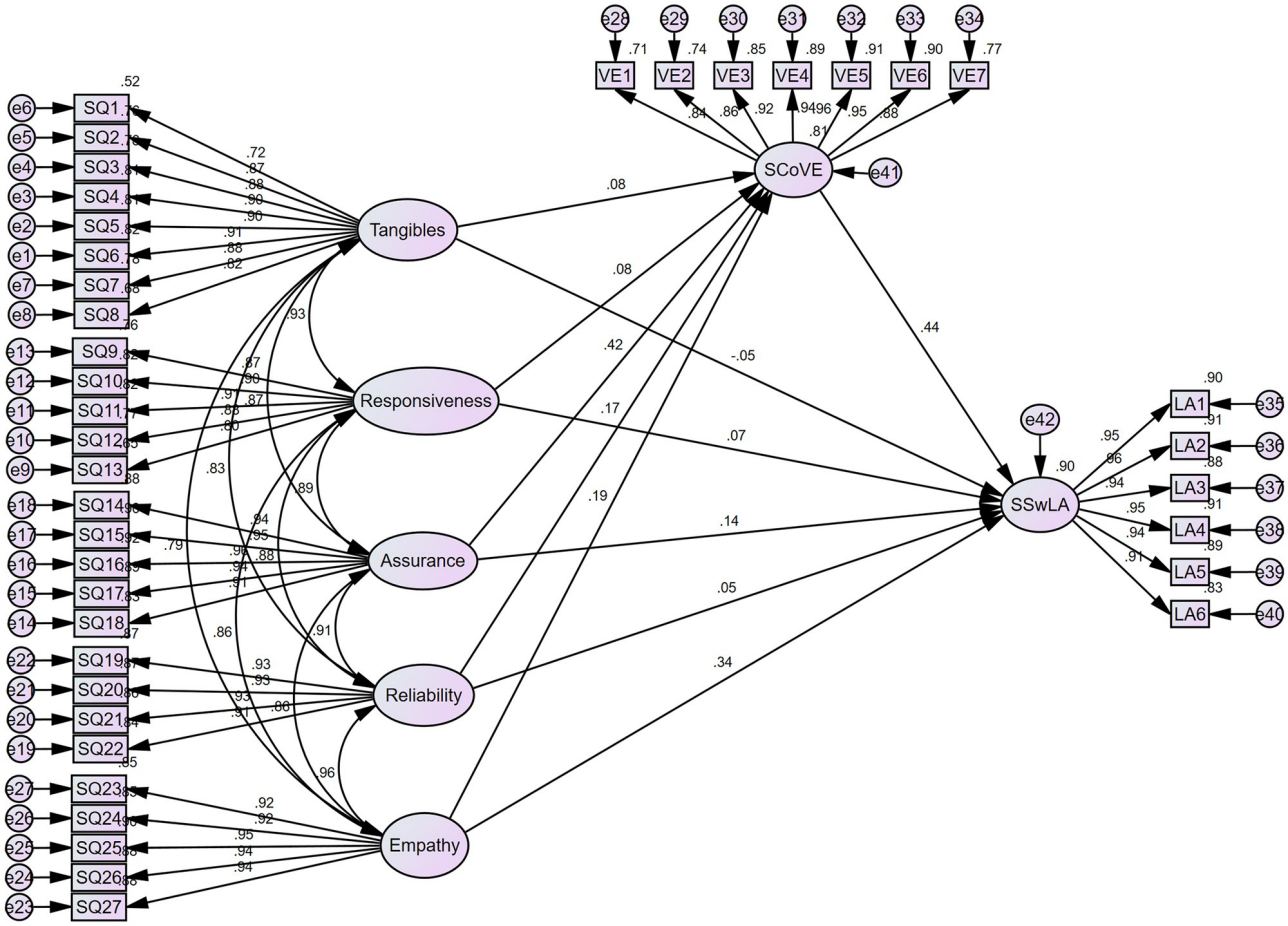
Final model with standardized path coefficients (Model 2). This figure represents the output from structural equation modeling (SEM) conducted using AMOS V.26 software, illustrating standardized path coefficients between variables.

All statistically non-significant paths were then deleted in Model 2. As shown in Fig 3, students’ satisfaction with learning achievements was positively predicted by four factors in the SPoSSQ (responsiveness, assurance, reliability, and empathy) and their perceptions of vocational education, but negatively predicted by the tangible concept. At the same time, all concepts in SPoSSQ had a positive contribution to SCoVE, suggesting that students who were more satisfied with their school’s service quality were more likely to have a positive attitude toward vocational education.

### Meditation analysis

A bootstrapping analysis was conducted to assess the significance of the mediating effect in Model 2. Table 5 displayed the findings, indicating that the impact of SPoSSQ on SSwLA was mediated by SCoVE. Specifically, the study tested H2, which posited a positive association between SPoSSQ and SSwLA. The results showed significant positive associations between the responsiveness, assurance, reliability, and empathy factors in SPoSSQ and SSwLA, as well as a significant negative association between tangibles and SSwLA. Consequently, Hypothesis 2 was not supported, due to the existence of this negative association. The bootstrapping analysis revealed significant indirect effects in Model 2, with changes that were observed and showed greater degrees of significance without any zero between the limits. Therefore, the study had solid grounds to accept hypothesis 3.

**Table 5:** Standardized total, direct, and indirect effects and 95% confidence intervals.

Path	β	Lower	Upper
Direct effect			
Tangibles → SSwLA	-.044	-.078	-.011
Responsiveness → SSwLA	.066	.024	.109
Assurance → SSwLA	.136	.093	.183
Reliability → SSwLA	.050	-.031	.129
Empathy → SSwLA	.352	.285	.420
Mediating effect			
Tangibles → SCoVE → SSwLA	.032	.014	.051
Responsiveness → SCoVE→ SSwLA	.035	.012	.059
Assurance → SCoVE→ SSwLA	.183	.156	.212
Reliability → SCoVE → SSwLA	.077	.036	.120
Empathy → SCoVE→ SSwLA	.085	.052	.119

## Discussion

The purpose of this research was to investigate the perceptions of students regarding the quality of school services and examine how these perceptions, along with their conceptions of vocational education, influence their satisfaction with learning in Chinese vocational education settings. The study demonstrated that students’ perceptions of vocational education play a significant mediating role in the link between perceived service quality and satisfaction with learning outcomes. This finding indicates that enhancing students’ perceptions of service quality can directly improve their satisfaction with learning achievements. According to the results, Chinese high school students in vocational education rated their school’s service quality from five aspects, which is similar to what other studies have found about the application of the service quality model in the educational sector [71–74]. The present study provides evidence for the suitability of the SERVPERF scale with its five dimensions for measuring service quality in China’s education sector. This finding is consistent with previous research, such as El-Hilali et al. [25] and Hussain and Birol [22], which affirm the SERVPERF scale’s applicability in educational contexts. However, our study diverges from Uppal et al. [26] and He [28] indicating that the dimensional structure of service quality may vary across contexts. This discrepancy underscores the necessity of contextualizing service quality models to specific educational environments, a theme echoed in our results and highlighted by the unique impact of different service quality dimensions on student satisfaction in the Chinese vocational education system. Literature review shows that although the service quality model formulated by Parasuraman et al. [20] is widely used, it is necessary to customize service quality evaluation to the situation because there is no universal set of dimensions and items that can be employed to gauge service quality across all industries. Our study establishes the validity of the SERVPERF instrument, which was tailored for use in vocational education in China and based on five criteria. These findings reinforce previous research that confirmed the applicability of the SERVQUAL scale to a range of service contexts [75, 76].

The results of the study on students’ conceptions of vocational education suggest that Chinese secondary vocational students expressed contentment with their school’s administrative policies as well as the teaching competencies and attitudes of their instructors. The dimension of empathy garnered the highest level of satisfaction, followed by responsiveness, assurance, and reliability, while tangibles received the lowest rating. The observation of significant satisfaction with empathy in this research is consistent with previous literature emphasizing the critical role of teacher care and support as a key predictor of school satisfaction [77, 78]. This finding reflects the dedication of teachers in Chinese vocational schools towards recognizing the requirements of students and providing necessary support to foster their academic and personal development.

This study sheds light on the prevailing public perception of Vocational Education and Training (VET) in China, often relegated to a secondary option influenced by cultural, social, and institutional factors. The cultural landscape, with its deep-rooted preference for academic education over vocational pathways, significantly shapes these perceptions [79]. This bias is reflected in the rural-urban divide concerning educational aspirations, highlighting the enduring challenge of transforming societal norms that have long favored academic pathways [80]. Despite concerted governmental efforts to reposition vocational education through policy reforms and public campaigns [81], changing entrenched societal perceptions has proven to be a complex task, marked by the intricate interplay of cultural values and educational policy. The societal stigma attached to vocational pathways, frequently perceived as inferior to academic routes, further complicates this picture. This stigma, bolstered by an exam-oriented education system [82] and the competitive pressures it engenders, distorts the perceived value and appeal of vocational education. The implications of this are profound, with societal perceptions being shaped by an uneven distribution of resources and the high stakes of college entrance examinations, which cast vocational education in an unfavorable light. On the institutional front, measures such as the “Vocational Education Law” represent governmental efforts to diminish the gap between vocational and academic education by advocating for higher quality standards and closer ties with industry [83]. Nonetheless, the persistence of disparities in funding, resources, and societal recognition continues to influence students’ perceptions of the value of vocational education negatively. Moreover, the curriculum’s emphasis on practical skills [84], aimed at equipping students for specific career paths, may be perceived as restrictive, especially within a culture that traditionally prizes academic achievement. To improve the perception and quality of vocational education in China, a comprehensive strategy is essential. This strategy must encompass not only government and institutional initiatives but also a shift in societal attitudes toward vocational education. Acknowledging the inherent value of vocational training and its contributions to personal development and the economy is crucial for overcoming cultural biases and elevating the status of vocational education.

The results of the mediation analysis revealed that Chinese secondary vocational students’ satisfaction with their learning achievements was influenced by their perceptions of school service quality and their conceptions of vocational education. It was found that students who were satisfied with their school’s service quality (tangibles, responsiveness, reliability, assurance, and empathy) and had favorable views toward vocational education were likely to be pleased with their learning outcomes in vocational schools. While students’ satisfaction with their schools’ responsiveness, assurance, and empathy influenced how satisfied they were with their academic performance, the tangibles factor had a direct negative impact on their learning satisfaction. This aligns with the findings of Magasi et al. [85], who noted that responsiveness, assurance, and empathy are essential for fostering satisfaction within higher education contexts in Africa. Interestingly, the reliability factor showed a minimal correlation with academic satisfaction in this study, contrasting with Darawong and Widayati [86]’s research, which highlighted reliability as a key driver of student satisfaction in online learning environments in Thailand. This divergence might stem from differences in service contexts, cultural nuances, and demographic characteristics of the study populations. Additionally, integrating psychological perspectives, it is evident that students’ learning satisfaction is closely tied to their personality traits and intrinsic motivations [87–89], suggesting that both contextual and individual factors significantly shape educational experiences. This underscores the importance of a tailored approach in vocational education and training (VET) programs, where recognizing and accommodating the diverse personalities and motivational drivers of students can significantly enhance service quality and satisfaction. By creating an educational environment that fosters social interaction, recognizes achievements, and encourages active knowledge-seeking, VET programs can stimulate students’ intrinsic motivation. This not only aligns with their individual needs and preferences but also bolsters their perception of vocational education as a rewarding and engaging pathway.

This study uncovers a noteworthy negative relationship between tangible elements and academic satisfaction in the context of Chinese vocational education. This contradicts prior research by El-Hilali et al. [25], Magasi et al. [85] and Gregory [90], which emphasize the significance of tangibles in shaping students’ satisfaction in higher education and bolstering an organization’s image. The current finding proposes that intangibles hold more weight in influencing students’ satisfaction. This discrepancy might be explained by the observed underutilization of digital resources and the lack of teacher training for effectively employing new training facilities, playgrounds, and laboratories [91]. Such a scenario leads students to perceive that increased spending on physical infrastructure negatively affects the allocation of resources towards curriculum development, teacher preparation, and instructional material management, which in turn detracts from their educational achievements. It is within this context that the role of technology in education becomes critically important [39]. By effectively harnessing digital tools and platforms [39], educational institutions can bridge the gap identified in this study, shifting focus from merely enhancing physical infrastructure to fostering an environment where technology acts as a cornerstone for improving student satisfaction through increased engagement, accessibility, and personalized learning experiences. This transition emphasizes the need to recalibrate resource allocation towards integrating technology in teaching and learning processes, marking a significant step towards addressing the concerns raised by the current.

This study offers a novel addition to the current body of research on students’ perceptions of vocational education, the quality of school services, and their satisfaction with their educational experiences. The results indicate that students’ conceptions of vocational education play a mediating role in the impact of school service quality on learning satisfaction. These findings highlight the importance of students’ perspectives on vocational education and suggest that long-standing stereotypes and inherent views on vocational education in Chinese society can be detrimental to students’ learning outcomes in vocational education, ultimately impacting their employability and the development of vocational education in China. Importantly, students’ conceptions of vocational education emerge as the strongest contributor to their learning outcomes, capable of dispelling their skepticism regarding their school’s substantial investment in hardware equipment and structures. These results suggest that enhancing students’ conceptions of vocational education could be an effective strategy to improve vocational education quality and increase student satisfaction in China.

## Implication and limitations

### Theoretical implication

This study significantly advances the field of vocational education by integrating service quality theory and models, particularly within the Chinese context. It applies the SERVPERF model, traditionally used in service industries, to vocational education. This innovative adaptation extends the theoretical application of service quality models by examining how specific dimensions—responsiveness, assurance, reliability, empathy, and tangibles—affect student satisfaction. By employing service quality models to systematically evaluate student perceptions, the study not only pinpoints areas where vocational education services excel but also identifies areas needing improvement, thereby guiding targeted enhancements. This is particularly vital in China, where there is a robust push for educational reform and an increasing demand for high-quality vocational training. Furthermore, the study enriches theoretical discussions by identifying students’ conceptions of vocational education as a mediating factor in the relationship between perceived service quality and learning satisfaction. This finding highlights the critical role of cognitive and affective factors in educational outcomes and suggests a more dynamic interplay between service delivery and educational perception than previously recognized. By emphasizing the importance of aligning educational practices with students’ conceptual frameworks, the study contributes valuable insights into how educational policies and practices can be adapted to meet the needs of vocational students more effectively.

### Practical implication

This study offers critical insights for both educational leaders and parents of secondary vocational students, emphasizing the need to improve educational service quality and reinforce trust in vocational education as key to enhancing student satisfaction with their learning experiences. For administrators of secondary vocational schools, it is essential to prioritize upgrades to facilities and instructional resources, and to focus on developing teachers’ communication abilities and teaching skills. Encouraging teachers to employ strategies that support students’ basic psychological needs for autonomy, competence, and relatedness, as outlined by Self-Determination Theory, is vital [49]. This includes providing choices, offering optimal challenges with constructive feedback, fostering a sense of belonging, setting fair expectations, engaging students actively, presenting content enthusiastically, and avoiding undermining behaviors [49]. Moreover, embedding practical skills development and reciprocal learning into Vocational Education and Training (VET) programs is crucial for fostering a supportive and interactive learning environment [92, 93]. This approach not only aligns with the Chinese government’s directive to ensure parity between general and vocational high schools but also holds significant implications for parents aiming to boost their children’s educational satisfaction. By encouraging confidence in the standards and effectiveness of vocational education, parents can play a pivotal role in elevating their children’s academic success within these programs.

### Limitations

The present study has several limitations that should be considered when interpreting its findings. Firstly, the research was conducted solely in a single city’s secondary vocational schools, which could limit the generalizability of the results to other regions or contexts. Therefore, future research could expand the scope of investigation by increasing the sample size and incorporating new variables to improve the explanatory power of the model. Additionally, future studies could examine the moderating effects of individual differences, cultural factors, and institutional characteristics on the relationships among students’ perceptions of vocational education, school service quality, and satisfaction with educational achievement. Such research would enhance our understanding of the intricate dynamics involved in vocational education and inform policy and practice in the field.

## Conclusion

This study revealed that the four SPoSSQ factors—responsiveness, assurance, reliability, empathy—as well as their perceptions of vocational education, positively influenced students’ satisfaction with learning achievements. In contrast, the tangibles aspect of SPoSSQ was found to negatively impact students’ learning satisfaction. Furthermore, every aspect in SPoSSQ favorably influenced students’ perceptions of vocational education (SCoVE). This shows that students who were more satisfied with their school’s service quality were more likely to be motivated about vocational education. It emphasizes the importance of addressing negative stereotypes about vocational education in China to enhance student learning satisfaction. Building on these insights, it is recommended that policymakers and educators in vocational education devise targeted interventions to reshape perceptions and counteract prevailing stereotypes. Specifically, developing comprehensive awareness campaigns and curriculum reforms that highlight the real-world applicability and success stories of vocational training can significantly alter student perceptions. These strategies not only aim to improve student satisfaction but also seek to elevate the standing of vocational education within the broader educational landscape. Overall, this study contributes to understanding the significance of perceptions in educational satisfaction and advocates for positive representations of vocational education to foster trust and confidence among students.

## Supporting information

S1 Appendix (DOCX)
