# Supplementary material for: Perceived school service quality and vocational students’ learning satisfaction: Mediating role of conceptions of vocational education
Source: PLoS One. 2024 Aug 21;19(8):e0307392. doi: 10.1371/journal.pone.0307392 (PMC11338464; doi:10.1371/journal.pone.0307392)
Supplement: S1 Appendix — (DOCX) [file pone.0307392.s001.docx]

**S1 Appendix. The Factors and Items in SPoSSQ, SSwLA and SCoVE.**

| **Items** | |
| --- | --- |
| **SPoSSQ: Students’ Perceptions of School Service Quality Scale** | |
|  | Tangibles |
| SQ1 | SQ1: The school's location facilitates convenient commuting. |
| SQ2 | The campus layout and environment are conducive to learning. |
| SQ3 | Access to learning resources (library, electronic resources) meets my academic needs. |
| SQ4 | The infrastructure (teaching buildings, dormitories, canteens, sports fields, libraries, laboratories, computer classrooms, practical training equipment) is well-maintained. |
| SQ5 | Classroom teaching equipment and the digital learning environment (multimedia equipment, etc.) enhance the learning experience. |
| SQ6 | Practical teaching facilities adequately support hands-on learning. |
| SQ7 | The school premises are clean, and medical services and living guarantees are satisfactory. |
| SQ8 | The food provided is varied, nutritious, safe, and hygienic. |
|  | Responsiveness |
| SQ9 | The academic atmosphere promotes learning discipline. |
| SQ10 | School systems (rules, rewards) are applied fairly to all students. |
| SQ11 | The school promptly addresses students' issues and suggestions. |
| SQ12 | Communication between school/teachers and my parents occurs frequently. |
| SQ13 | The school offers adequate financial aid and scholarships. |
|  | Assurance |
| SQ14 | Practical training courses are sufficiently balanced with theoretical learning. |
| SQ15 | Participation in vocational training programs that integrate industry and education is encouraged and frequent. |
| SQ16 | Vocational skills training sessions are frequent and relevant. |
| SQ17 | Special courses and activities organized by the school meet my learning interests. |
| SQ18 | The variety of club activities or practical activities supports my personal development. |
|  | Reliability |
| SQ19 | Teachers possess strong teaching abilities, reflected in their lecture content and methods. |
| SQ20 | Teachers correct homework with attention and seriousness. |
| SQ21 | Teachers offer after-school tutoring support. |
| SQ22 | Teachers demonstrate a positive work attitude. |
|  | Empathy |
| SQ23 | Teachers show a caring attitude towards students and their parents. |
| SQ24 | Teachers treat all students equally and with respect. |
| SQ25 | Teachers are serious and helpful when answering questions. |
| SQ26 | Teachers provide assistance when I face difficulties. |
| SQ27 | Teachers encourage me in both my studies and personal life. |
| **SSwLA: Students’ Satisfaction with Learning Achievements Scale** | |
| LA1 | I have observed a change in my attitude towards learning throughout my academic life at school. |
| LA2 | I have noticed enhancements in my learning methods and habits during my time at school. |
| LA3 | My physical fitness has improved as a result of my academic life at school. |
| LA4 | I have experienced personal growth throughout my academic life at school. |
| LA5 | My academic experiences at school have increased my confidence about life. |
| LA6 | LA6: My approach to handling setbacks is now more positive and optimistic. |
| **SCoVE: Students’ Conceptions of Vocational Education** | |
| VE1 | I have a positive attitude towards vocational education. |
| VE2 | I agree with the policy of directing students into either regular high schools or vocational schools after middle school. |
| VE3 | I believe vocational education offers significant educational advancement opportunities. |
| VE4 | I am optimistic about the future job prospects within my field of study due to my vocational education. |
| VE5 | I view the career development prospects for technically skilled professionals positively. |
| VE6 | I intend to pursue a career that is related to my major in the future. |
| VE7 | I support national policies that promote the development of vocational education. |
